# GABA_A_ receptors in epilepsy: Elucidating phenotypic divergence through functional analysis of genetic variants

**DOI:** 10.1111/jnc.15932

**Published:** 2023-08-24

**Authors:** Nathan L. Absalom, Susan X. N. Lin, Vivian W. Y. Liao, Han C. Chua, Rikke S. Møller, Mary Chebib, Philip K. Ahring

**Affiliations:** ^1^ School of Science University of Western Sydney Sydney New South Wales Australia; ^2^ Brain and Mind Centre, School of Medical Sciences, Faculty of Medicine and Health The University of Sydney Sydney New South Wales Australia; ^3^ Brain and Mind Centre, Sydney Pharmacy School, Faculty of Medicine and Health The University of Sydney Sydney New South Wales Australia; ^4^ Department of Epilepsy Genetics and Personalized Medicine The Danish Epilepsy Centre, Filadelfia Dianalund Denmark; ^5^ Department of Regional Health Research University of Southern Denmark Odense Denmark

**Keywords:** ACMG, epilepsy, GABA, neurotransmission

## Abstract

Normal brain function requires a tightly regulated balance between excitatory and inhibitory neurotransmissions. γ‐Aminobutyric acid type A (GABA_A_) receptors represent the major class of inhibitory ion channels in the mammalian brain. Dysregulation of these receptors and/or their associated pathways is strongly implicated in the pathophysiology of epilepsy. To date, hundreds of different GABA_A_ receptor subunit variants have been associated with epilepsy, making them a prominent cause of genetically linked epilepsy. While identifying these genetic variants is crucial for accurate diagnosis and effective genetic counselling, it does not necessarily lead to improved personalised treatment options. This is because the identification of a variant does not reveal how the function of GABA_A_ receptors is affected. Genetic variants in GABA_A_ receptor subunits can cause complex changes to receptor properties resulting in various degrees of gain‐of‐function, loss‐of‐function or a combination of both. Understanding how variants affect the function of GABA_A_ receptors therefore represents an important first step in the ongoing development of precision therapies. Furthermore, it is important to ensure that functional data are produced using methodologies that allow genetic variants to be classified using clinical guidelines such as those developed by the American College of Medical Genetics and Genomics. This article will review the current knowledge in the field and provide recommendations for future functional analysis of genetic GABA_A_ receptor variants.

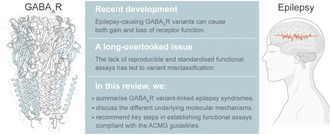

## Graphical abstract

GABA_A_ receptors mediate inhibitory neurotransmission and are key for maintaining the excitatory/inhibitory balance throughout the brain. Genetic variants in GABA_A_ receptor subunits were among the earliest to be associated with epilepsy, and several hundreds of variants have since been linked to epileptic syndromes. Until recently, these genetic variants were believed to cause only loss of function leading to diminished inhibition in the brain, however, recent data reveal that this is incorrect. Here, we describe the evolution of GABA_A_ receptor variant discoveries, how historical functional analysis failed to explain clinical phenotypes and discuss the need for a new level of rigor in the scientific community when assessing genetic variants.

**Abbreviations**
ACMG: American College of Medical Genetics and Genomics
ASM: anti‐seizure medication
CAE: childhood absence epilepsy
DEE: developmental and epileptic encephalopathy
DS: Dravet syndrome
EEG: electroencephalogram
ER: endoplasmic reticulum
FS: febrile seizures
GABA_A_
: gamma‐aminobutyric acid type A
GABA_B_
: gamma‐aminobutyric acid type B
GAT‐1: GABA transporter 1
GEFS+: genetic epilepsy with febrile seizures plus
GGE: genetic generalised epilepsy
gnomAD: genome aggregation database
GTCA: epilepsy with generalised tonic–clonic seizures alone
GTCS: generalised tonic–clonic seizures
JAE: juvenile absence epilepsy
JME: juvenile myoclonic epilepsy
KCC2: K^+^/Cl^−^ cotransporter 2
LGICs: ligand‐gated ion channels
LGS: Lennox–Gastaut syndrome
PET: positron emission tomography
PV: parvalbumin
RELN: reelin
SOM: somatostatin
VIP: vasoactive intestinal peptide

## 1 INTRODUCTION

Epilepsy is characterised by a predisposition to a hyper‐synchronised wave of neuronal discharge (Fisher et al., 2014). The clinical phenotypes of patients with epilepsy can range from treatable epilepsies in normally developed individuals to severe developmental and epileptic encephalopathies (DEEs) with age‐specific presentations and comorbidities (Hirsch et al., 2022). Patients with DEE are often pharmacoresistant and exhibit comorbidities including developmental delay and intellectual deficits, movement disorders and neuropsychiatric symptoms such as autism spectrum disorder. The underlying pathological mechanism of epilepsy is complex, but it is known that genetic variants play a key role in many cases. These genetic variants can be either inherited or de novo meaning that they are seen for the first time in a patient but not in the parents. Notable ion channel genes associated with epilepsy include GABA_A_ receptor subunits (GABR*), voltage‐gated sodium and calcium channels (e.g., *SCN1A* and *CACNA1A*) and potassium channels (e.g., *KCNQ2*) (Simkin et al., 2022).

GABA_A_ receptors mediate inhibitory neurotransmission and are key for maintaining the excitatory/inhibitory balance throughout the brain. Due to this importance for brain homeostasis, the GABAergic system can exert both epilepsy‐suppressing and epilepsy‐promoting effects. Drugs that target GABA_A_ receptors such as benzodiazepines have been used as anti‐seizure medications (ASMs) for decades and typically act to increase GABAergic function thereby reducing brain excitations levels (Kienitz et al., 2022; Treiman, 2001). Furthermore, genetic variants in GABA_A_ receptor subunits were among the earliest to be associated with epilepsy, and several hundreds of variants have been linked to epileptic syndromes since the early genetic discoveries (Absalom et al., 2022; Ahring et al., 2022; Cossette et al., 2002; Hernandez et al., 2019; Johannesen et al., 2016; Maljevic et al., 2019; Møller et al., 2017; Niturad et al., 2017).

In recent years, the number of epilepsy‐associated GABA_A_ receptor variants discovered has grown at a seemingly exponential rate that surpasses the testing capacity of conventional academic research laboratories. For this reason, it has become common in the field to infer the potential pathogenicity of a set of variants based on functional analysis performed on selected few variants (El Achkar et al., 2021; May et al., 2018). However, this is not a viable long‐term strategy as it increases the risks of misinterpreting variants, leading to misdiagnosis and inappropriate treatment. Hence, to progress the field towards precision therapy, standardised approaches for functional analysis and integration with clinical guidelines are needed. This article will review the clinical manifestations of genetic GABA_A_ receptor variants, the early‐stage efforts undertaken by research laboratories to determine their functional consequences and the complex challenges faced in the field. A foundation will be provided to understand how variants can impact receptor function. Additionally, it will provide recommendations for conducting future laboratory efforts to generate functional data that align with clinical guidelines to determine variant pathogenicity rapidly and accurately.

## 2 GABAERGIC INTERNEURONS AND GABA_A_ RECEPTORS

**FIGURE 1:**
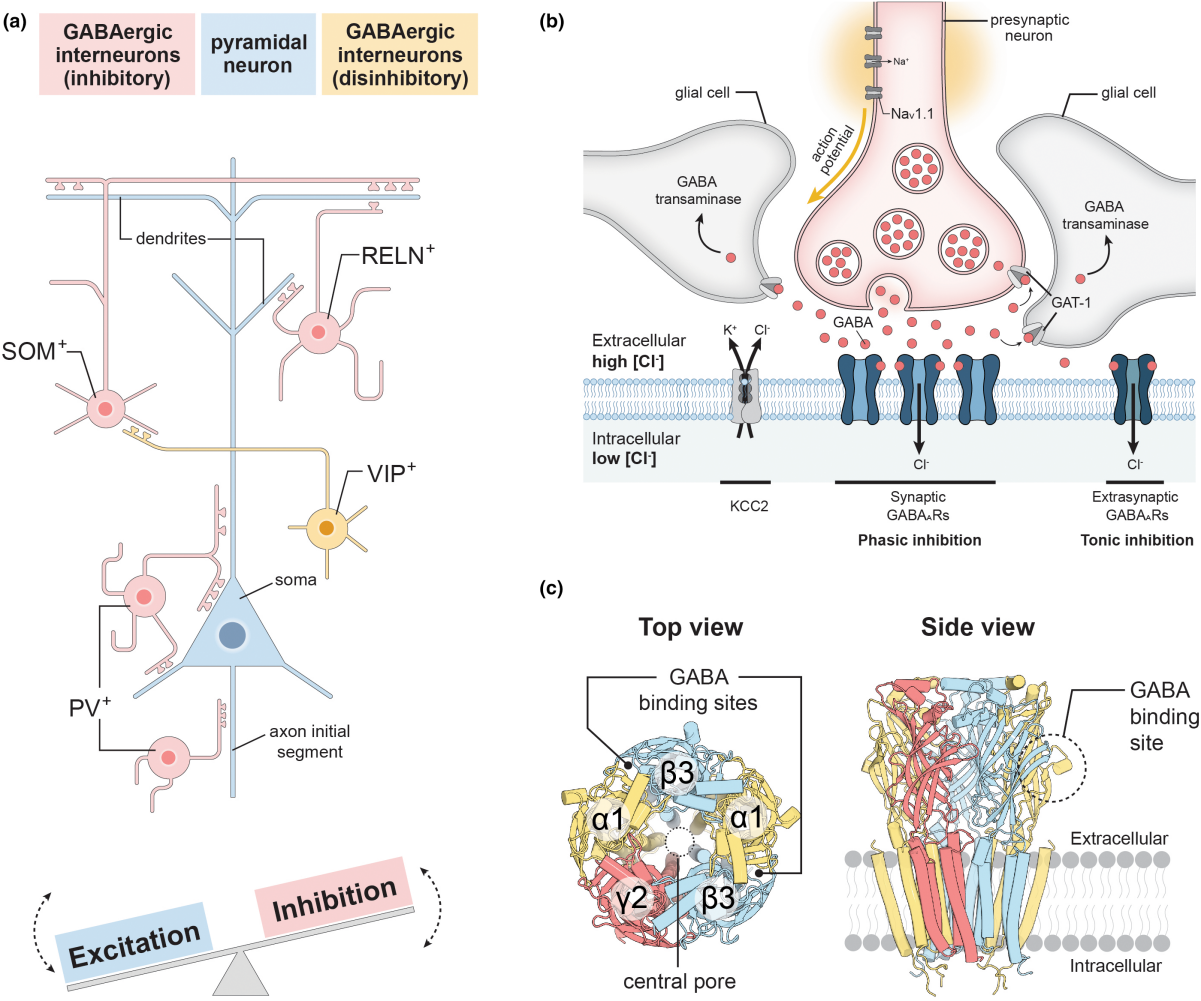
GABAergic interneurons and GABA_A_ receptors. (a) Cartoon illustrating the connectivity of principal cells (blue) and different types of GABAergic interneurons (pink and orange), including parvalbumin‐expressing (PV^+^), somatostatin‐expressing (SOM^+^), vasoactive intestinal peptide‐expressing (VIP^+^) and reelin‐expressing (RELN^+^) neurons. Interneurons play a vital role in maintaining the excitation/inhibition balance by providing inhibitory inputs that target different regions of a postsynaptic neuron, which can either be a pyramidal cell or interneuron. Figure adapted from (Tang et al., 2021). (b) GABA release, reuptake and degradation: Presynaptic neuron releases GABA which activates two types of GABA_A_ receptors: synaptic and extrasynaptic GABA_A_ receptors, responsible for phasic and tonic inhibition, respectively. To ensure the termination of GABAergic transmission, GABA transporters type 1 (GAT‐1) facilitate the reuptake of GABA into the presynaptic neuron and neighbouring glial cells. In glial cells, GABA is broken down by GABA transaminase within the mitochondria. The potassium‐chloride cotransporter 2 (KCC2) plays a crucial role in regulating intracellular chloride concentration, which is essential for the effectiveness of GABAergic inhibition. (c) Cryo‐EM structure of the α1β3γ2L GABA_A_ receptors (PDB 6HUO) demonstrating a 2α:2β:1γ stoichiometry, γ‐β‐α‐β‐α arrangement (read counter‐clockwise when viewed from the extracellular side) and the orthosteric GABA binding sites.

In the brain, excitatory and inhibitory neurons form intricately interconnected circuits coordinating complex cognitive processes. Pyramidal neurons are the most common excitatory neurons in the mammalian cerebral cortex, using the neurotransmitter glutamate to stimulate neuronal activity (Bekkers, 2011; Merchán‐Pérez et al., 2009; Spruston, 2008) (Figure 1a). Meanwhile, inhibitory interneurons release the neurotransmitter GABA to coordinate circuit activity in a highly complex manner. Specifically, GABAergic inhibition modulates gain or sensitivity of neural circuits (Katzner et al., 2011), shapes spatiotemporal dynamics of excitatory signals (Villar et al., 2021), tunes the stimulus selectivity of individual neurons (Priebe & Ferster, 2008) and synchronises neuronal firing which enables neurons to cooperate to convey signals to common downstream targets (Mann & Paulsen, 2007). These GABAergic interneurons have diverse anatomical, molecular and physiological features (Huang & Paul, 2019; Topolnik & Tamboli, 2022) and can be categorised into four main classes based on the specific genetic markers they express: the calcium‐binding protein parvalbumin (PV), the neuropeptide somatostatin (SOM), vasoactive intestinal peptide (VIP) and the glycoprotein reelin (RELN) (Tang et al., 2021; Wamsley & Fishell, 2017). PV^+^ neurons directly target the soma and the axon initial segment of pyramidal neurons to reduce neuronal firing. SOM^+^ and RELN^+^ neurons, on the other hand, target mainly the distal dendrites to regulate the integration of excitatory synaptic inputs. By contrast, VIP^+^ neurons preferentially inhibit SOM^+^ neurons, leading to disinhibition of pyramidal neurons. Therefore, by innervating different cellular compartments of the pyramidal neuron, GABAergic neurons fine‐tune excitability of pyramidal neurons across space and time, which is crucial in the maintenance of circuit‐level excitation/inhibition balance (Cardin, 2018; Isaacson & Scanziani, 2011; Tremblay et al., 2016). Given its fundamental regulatory roles, it is perhaps not surprising that GABAergic inhibition is implicated in various physiological processes such as neuronal development (Tang et al., 2021), learning (Ferguson & Cardin, 2020; Ren et al., 2012), memory (McNally et al., 2008; Topolnik & Tamboli, 2022), circadian rhythm (Gillespie et al., 1997; Mintz et al., 2002; Ralph & Menaker, 1989; Topolnik & Tamboli, 2022), sleep (Saper et al., 2005) and pain (Enna & McCarson, 2006; Moore et al., 2002; Munro et al., 2008), despite comprising only 20%–30% of all neurons (Enna & McCarson, 2006; Mody & Pearce, 2004).

### 2.1 GABA‐evoked responses

When GABA is released into the synaptic cleft, it binds and activates two distinct types of receptors known as the ionotropic GABA_A_ and the metabotropic GABA_B_ receptors. GABA_A_ receptors are the most widespread GABA receptors in the CNS, facilitating fast inhibitory responses (<10 ms) in the regulation of neuronal excitability. These receptors are highly concentrated on postsynaptic neuronal membrane, but they are also expressed extrasynaptically (Figure 1b). Synaptic and extrasynaptic GABA_A_ receptors regulate circuit activity through two distinct modes of inhibition: phasic and tonic inhibition, respectively (Joo et al., 2014; Lee & Maguire, 2014). Activation of presynaptic GABA_B_ receptors triggers different downstream effector mechanisms that result in the inhibition of GABA release from GABAergic neurons (GABA_B_ receptors are outside the scope of this review – for a recent review see Sanchez‐Vives et al., 2021). A classical view of cortical GABAergic inhibition begins with firing of an action potential at a presynaptic neuron, mediated by sodium influx through voltage‐gated sodium channels (e.g., Na_V_1.1 channels) (Figure 1b). GABA is released into the synaptic cleft where it binds to GABA_A_ receptors at the postsynaptic neuron that open, allowing chloride to pass into the cell. Away from the synapse, lower concentrations of GABA activate extrasynaptic GABA_A_ receptors. At mature neurons, an inhibitory chloride gradient is typically maintained by K^+^/Cl^−^ cotransporter 2 (KCC2) channels transporting chloride out of the cell. In contrast, Na‐K‐Cl cotransporter 1 (NKCC1) maintains a depolarising chloride gradient in immature neurons (for a recent review discussing transporter function see Bryson et al., 2023). GABA is recycled via GABA transporters including GABA transporter 1 (GAT‐1) and broken down by GABA transaminase. A wealth of evidence implicates these processes of the GABAergic system with epilepsy, where drugs that increase either extracellular GABA concentrations or the activity of GABA_A_ receptors are effective ASMs, and genetic variants that lead to dysfunctional GABA signalling are associated with various forms of epilepsy and neurodevelopmental disorders (Bryson et al., 2023).

### 2.2 Structure and function of GABA_A_ receptors

GABA_A_ receptors are members of the Cys‐loop receptor superfamily of ligand‐gated ion channels (LGICs), which includes other prominent receptors such as the nicotinic acetylcholine, 5HT_3_ and glycine receptors (Alexander et al., 2017). Cys‐loop receptors share a common topology and are composed of five membrane‐spanning subunits in a symmetrical or pseudo‐symmetrical arrangement around a central pore (Figure 1c) (Daniel & Öhman, 2009; Sine & Engel, 2006). A typical subunit consists of approximately 500 amino acids split almost equally into a large N‐terminal extracellular domain that forms the ligand binding site and a transmembrane domain that forms the ion channel pore.

Most GABA_A_ receptors are pseudosymmetrical, as the pentameric receptor complex is assembled from different types of subunit proteins. A total of 19 genes code for the subunits of the GABA_A_‐receptor family termed α1‐6, β1‐3, γ1‐3, δ, ϵ, π, θ, and ρ1‐3. The genes encoding most of these subunits are clustered on chromosomes 4, 5, 15 and X (Simon et al., 2004). The three ρ subunits were historically referred to as GABA_C_ receptor subunits and differ by their ability to assemble as homomers and their inability to form functional receptors other than in combination with other ρ subunits (Enz, 2001).

GABA_A_‐receptor subunits can mix and match to form a wealth of receptor subtypes. While this could theoretically give a staggering number of potential receptor combinations, only a limited number (Enna & McCarson, 2006; Enz, 2001; Essajee et al., 2022; Eugène et al., 2007; Fedi et al., 2006; Ferguson & Cardin, 2020; Fisher, 2004; Fisher et al., 2014; Frugier et al., 2007; Fu et al., 2022; Gielen et al., 2012) of subtypes are believed to be naturally expressed in the mammalian brain (Mortensen et al., 2011; Olsen & Sieghart, 2009; Richard & Werner, 2008; Whiting, 2003). The most common isoforms are composed of two α's, two β's and either a γ2 or δ subunit, and of these three receptor subtypes, α1β2/3γ2, α2β2/3γ2 and α3β2/3γ2, are believed to constitute the vast majority (~80%) of receptors in the brain. The different subunit compositions result in an array of receptor characteristics, including GABA sensitivity, conductance and desensitisation, and different spatiotemporal distributions that are important to regulate neurophysiological responses such as movement and learning (Maljevic et al., 2006; Sigel & Ernst, 2018). Fast, phasic inhibition is mediated by synaptic GABA_A_ receptors, which respond to high concentration of synaptically released GABA (Lee & Maguire, 2014). Subunits that are involved in phasic inhibition typically include α1‐3, β2‐3 and γ2. Persistent, tonic inhibition is mediated by GABA_A_ receptors in the extrasynaptic spaces, which respond to consistent, low concentrations of GABA. Extrasynaptic GABA_A_ receptors typically contain α4‐6 and/or δ subunits (Glykys & Mody, 2007).

## 3 GABA_A_ RECEPTORS AND EPILEPSY SYNDROMES

Considering the significant role of GABA_A_ receptors in regulating the excitation/inhibition balance of the central nervous system, it is not surprising that genetic variants of GABA_A_ receptor genes are associated with epilepsy disorders. What is perhaps more surprising is that variant‐associated epilepsy ranges from mild genetic generalised epilepsies to severe DEEs, particularly since most variants in the GABA_A_ receptor genes follow an autosomal dominant pattern. This means that patients are heterozygous for the variant, possessing a mutant allele and a normal functioning allele.

### 3.1 Generalised genetic epilepsies

Genetic generalised epilepsies are characterised by the presence of generalised seizure types including for instance absence, myoclonic or bilateral tonic–clonic seizures and generalised spike and wave discharges on the electroencephalogram (EEG). These include specific electro‐clinical syndromes such as childhood absence epilepsy (CAE) and juvenile absence epilepsy (JAE), juvenile myoclonic epilepsy (JME), epilepsy with generalised tonic–clonic seizures alone (GTCA) and genetic epilepsy with febrile seizures plus (GEFS+).

CAE and JAE are syndromes characterised by absence seizures and 2.5–4 Hz generalised spike‐wave discharges on the EEG. In most of the patients, absence seizures are provoked by hyperventilation. Most patients with CAE or JAE have normal development and intelligence. Others including those with very frequent seizures can have learning difficulties, and some may also have attention, concentration, and memory disorders (Hirsch et al., 2022). GABA_A_ receptor genetic variants associated with CAE and JAE have been identified in the *GABRA1* (Cossette et al., 2002; Maillard et al., 2022; Maljevic et al., 2006, 2019) and the *GABRG2* (Hernandez et al., 2023; Kananura et al., 2002; Koko et al., 2022; Marini et al., 2003; Reinthaler et al., 2015; Wallace et al., 2001) genes. Less commonly, absence seizures have been reported in *GABRB2* (Maillard et al., 2022) and *GABRB3* (Johannesen et al., 2022; Maljevic et al., 2019) genes. The majority of children with CAE respond well to ASMs and are seizure free by mid‐adolescence. Individuals with JAE usually also respond to treatment; however, JAE is considered a life‐long condition, which means that ASMs are needed to maintain seizure control (Hirsch et al., 2022).

Other genetic generalised epilepsy syndromes, including JME and GTCA, have been observed with variants in the *GABRA1* (Cossette et al., 2002; Maillard et al., 2022), *GABRB2*, *GABRB3* and *GABRG2* (Maillard et al., 2022) genes. These syndromes are commonly diagnosed during teenage years and characterised by myoclonic jerks, absence seizures, generalised tonic–clonic seizures (GTCS) and the presence of 3–5.5 Hz generalised spike‐wave discharges on the EEG. Most of these patients require long‐term treatment with ASMs but the majority achieve good seizure control (Hirsch et al., 2022).

### 3.2 Febrile seizures and GEFS+

Febrile seizures (FS) are seizures that occur in 2%–6% of young children, typically between the age of 6 months to 5 years, and often triggered by a fever (38°C or greater). The frequency of seizures can reduce with age and have generally subsided by 5–6 years of age (Smith et al., 2019). FS can be simple or complex, lasting for more than 15 minutes and associated with focal neurological findings. FS are relatively common in GABA_A_ receptor‐associated epilepsies caused by variants in *GABRA1* (Johannesen et al., 2016; Maillard et al., 2022; Zhang & Liu, 2022), *GABRB3* (Johannesen et al., 2022) or *GABRG2* (Audenaert et al., 2006; Hancili et al., 2014; Hung et al., 2017). The variants are often inherited but can also be de novo in some cases.

Although patients can initially present with FS, they may progress into more severe syndromes where treatment becomes more difficult. One such syndrome is GEFS+, a familial epilepsy syndrome group with autosomal dominant inheritance patterns (Johannesen et al., 2022; Maillard et al., 2022; Myers, 2022; Myers et al., 2017). Typically, seizures start between 3 months and 6 years of age triggered by fever and are generalised and short lasting for most patients. However, instead of the common FS syndrome where patients are likely to outgrow the fever‐induced seizures by the age of 6 years, patients with GEFS+ will continue to have febrile or afebrile types of seizures through life. A number of *GABRA1* (Maillard et al., 2022), *GABRB3* (Johannesen et al., 2022; Maillard et al., 2022) and *GABRG2* (Baulac et al., 2001; Johnston et al., 2014; Maillard et al., 2022; Marini et al., 2003; Tian et al., 2013; Yamamoto et al., 2019) variants have been described to cause GEFS+. Seizures are often well‐controlled in this group with valproate, a commonly used effective treatment (Johannesen et al., 2022; Maillard et al., 2022; Marini et al., 2003; Sun et al., 2008). In addition, intrafamilial variability has been described; thus, there may be GEFS+ families where some family members develop more severe forms of epilepsy such as myoclonic atonic epilepsy or Dravet syndrome.

### 3.3 Developmental and epileptic encephalopathies (DEEs)

DEE is a group of severe childhood epilepsy syndromes where developmental impairments are driven by both the underlying genetic cause and the frequent, poorly controlled epileptiform activity (Specchio et al., 2022). DEE is characterised by young age of seizure onset, pharmacoresistant epilepsy, developmental delay, and abnormal electroencephalographic findings (Lin et al., 2022; Mierzewska et al., 2021). Clinical manifestations of DEE are on a spectrum but may include varying degrees of neurodevelopmental impairment such as developmental delay and intellectual disability varying from mild to severe, microcephaly, movement disorders, hypotonia, autism spectrum disorders and behavioural difficulties (Essajee et al., 2022; Kang et al., 2021; Matricardi et al., 2020; Strzelczyk & Schubert‐Bast, 2022). Specific electroclinical syndromes classified as DEE include Dravet syndrome, Ohtahara syndrome, Lennox–Gastaut syndrome (LGS), West syndrome and epilepsy of infancy with migrating focal seizures.

DEEs of different types have commonly been reported to be associated with GABA_A_ receptor variants, including in *GABRA1* (Bai et al., 2019; Johannesen et al., 2016; Kodera et al., 2016; Maillard et al., 2022; Reyes‐Nava et al., 2020), *GABRA2* (Butler et al., 2018), *GABRA3* (Niturad et al., 2017), *GABRA5* (Butler et al., 2018), *GABRB2* (Maillard et al., 2022), *GABRB3* (Absalom et al., 2022; Bagnall et al., 2017; Hernandez, Zhang, et al., 2017; Johannesen et al., 2022; Liu et al., 2018; Møller et al., 2017; Štěrbová et al., 2018; Zhang et al., 2017), *GABRG2* (Komulainen‐Ebrahim et al., 2019; Maillard et al., 2022; Scheffer et al., 2023; Shen et al., 2017; Zaganas et al., 2021) and *GABRD* (Ahring et al., 2022) genes. The variants are rarely inherited and almost always occur de novo. Common comorbidities of GABA_A_ receptor‐associated DEEs include intellectual disability that range from mild to severe and include behavioural disorders. Severe movement disorders such as dystonia, dyskinesias or chorea are most frequently seen in *GABRB2* variants (Maillard et al., 2022) but have also been reported in *GABRB3* and *GABRG2* variants. Hypotonia is also commonly reported, and microcephaly is seen in the more severe cases of DEE. Seizure types can vary widely, but focal rather than generalised seizures are common in GABA_A_ receptor‐associated DEEs (Absalom et al., 2022). These DEEs are very difficult to manage, and treatments are often ineffective regardless of the subunit the variant is located in (Absalom et al., 2022; El Achkar et al., 2021; Maillard et al., 2022).

Dravet syndrome (DS) is a prominent form of DEE featuring febrile seizure onset at 3–5 months of age and progressive worsening of seizure frequency and severity. The epilepsy syndrome typically worsens between the ages of 1–5 years, with the patient starting to experience a multitude of other seizure types including myoclonic, atypical absence, hemiclonic, focal and long‐lasting bilateral tonic–clonic seizures (Wheless et al., 2020). Common comorbidities include developmental delay, intellectual disability, motor impairment and behavioural concerns (Lagae, 2021; Wirrell et al., 2022; Ziobro et al., 2018). While the most common cause of DS is loss‐of‐function *SCN1A* variants, comprising of up to >90% of patients, it has also been widely reported at both nonsense and missense variants in different GABA_A_ receptor subunits. It is relatively commonly reported at *GABRG2* variants (Harkin et al., 2002; Hernandez et al., 2021; Hernandez, Kong, et al., 2017; Hirose, 2006; Ishii et al., 2014; Maillard et al., 2022; Singh et al., 1999) and also, but less frequently at *GABRB3* variants (Johannesen et al., 2022; Møller et al., 2017) and *GABRA1* variants (Carvill et al., 2014). While ASMs can be effective at reducing seizure frequency, it is rare that seizure freedom is achieved even with polytherapy, and comorbidities are complicated and difficult to manage (Harkin et al., 2002; Hernandez et al., 2021; Maillard et al., 2022).

### 3.4 Summary

It is clear that patients with GABA_A_ receptor variants can display diverse phenotypes ranging from mild conditions to severe DEE. The common GABA_A_ receptor genes implicated are *GABRA1*, *GABRB2*, *GABRB3* and *GABRG2*. However, epilepsy syndromes are also seen with lower frequency in other GABA_A_ receptor genes such as *GABRA2, GABRA3, GABRA5* and *GABRB1*. This is fully consistent with the notion that α1β2/3γ2 receptors are the most common GABA_A_ receptor subtypes in the mammalian brain. While *GABRG2* variants are often associated with FS, suggesting some linkage between the variant gene and patient phenotype, few patterns have emerged to associate specific syndromes with groups of common subunit variants. A spectrum of mild as well as very severe phenotypes are observed for all GABA_A_ receptor genes; thus, it is important to uncover the underlying molecular and physiological processes responsible for these vastly different clinical presentations.

## 4 FIRST IDENTIFICATION AND FUNCTIONAL ANALYSIS OF EPILEPSY‐ASSOCIATED GABA_A_ RECEPTOR VARIANTS

### 4.1 The first familial *GABRG2* variants

The first definitive reports of epilepsy‐associated genetic GABA_A_ receptor variants were published concurrently, describing two familial *GABRG2* variants p.(Lys328Met) (Baulac et al., 2001) and p.(Arg82Gln) (Wallace et al., 2001). At the time of discovery, it was common in the scientific community to use a numbering system denoting the mature peptide sequence, and since the γ2 subunit has a predicted 39‐amino‐acid signal peptide, these variants are often referred to as p.(Lys289Met) and p.(Arg43Gln). The penetrance and clinical outcomes of the two variants differed within the families, with the p.(Lys328Met) variant displaying full penetrance of a GEFS+ syndrome phenotype in a limited cohort, and the p.(Arg82Gln) variant displaying roughly 75% penetrance with a mixture of CAE and FS (Baulac et al., 2001; Marini et al., 2003). As these variants are located on different regions in the receptor, an obvious question arises whether these variants cause distinct functional changes that are responsible for the differences in the clinical phenotypes.

Initial functional electrophysiological evaluation identified reduced GABA‐elicited maximum current amplitudes for the p.(Lys328Met) variant (Baulac et al., 2001) while receptors with the p.(Arg82Gln) variant were unresponsive to benzodiazepine modulation (Wallace et al., 2001). However, subsequent co‐immunoprecipitation and positron emission tomography (PET) studies demonstrated that the p.(Lys328Met) variant was expressed at the cell surface at a level similar to that of the wild‐type, while reduced surface expression and retention of receptor complexes in intracellular compartments was seen for the p.(Arg82Gln) variant (Fedi et al., 2006; Frugier et al., 2007). Although temperature‐dependent trafficking defects were proposed as a factor for both variants (Kang et al., 2006), none of these findings were replicated in a later study that instead identified increased synaptic decay rates consistent with impaired receptor gating (Eugène et al., 2007). While the functional data for these two variants thus yielded somewhat mixed results, the overall evaluation pointed to loss‐of‐function as the underlying pathophysiological mechanism, albeit it was not clear why the penetrance and clinical phenotypes differed.

### 4.2 *GABRG2* nonsense variants

Sequencing of the *GABRG2* gene in familial epilepsies quickly identified more variants, confirming the association between *GABRG2* variants and epilepsy syndromes. These included a nonsense variant *GABRG2* p.(Gln390*) (Harkin et al., 2002) and a splice‐site variant *GABRG2* c.(IVS6 + 2T>G) (Kananura et al., 2002), expanding the types of variants beyond the initial missense variants. A clear loss‐of‐function variant on the sequence alone, the truncated Gln390* protein was found to be efficiently translated, but assembled receptors were retained in the endoplasmic reticulum (ER) (Harkin et al., 2002; Kang et al., 2009). This caused a dominant‐negative effect that was speculated to underlie more severe forms of epilepsy than mere haploinsufficiency (Kang et al., 2009); however, the familial inheritance of this variant was complex. Some family members tested negative for the variant but reported FS or myoclonic‐astatic epilepsy, making the correlation of dominant negative variants with more severe epilepsies less clear‐cut (Harkin et al., 2002). The *GABRG2* c.(IVS6 + 2T>G) was also described as a dominant negative, with the altered splice site leading to a frameshift and ultimately truncated protein that formed receptors retained in the ER (Tian & Macdonald, 2012). Although the inheritance pattern was less complex for this variant, the affected patients reported relatively mild syndromes of CAE and FS.

### 4.3 Familial variants in other GABA_A_ receptor genes

Subsequent studies of familial epilepsies expanded the range of GABA_A_ receptor subunits containing genetic variants. Two *GABRD* variants, p.(Glu177Ala) and p.(Arg220His), were identified in families with FS and JME, respectively (Dibbens et al., 2004). Intriguingly, electrophysiological analysis revealed reduced currents of δ‐containing receptors for both variants despite the observation that the p.(Arg220His) variant was also found in the general population. A *GABRA1* p.(Ala322Asp) variant was identified in a family with JME with complete penetrance, and electrophysiological analysis demonstrated that the receptor gating properties were severely impaired, leading to a significant loss of GABA‐activated currents (Cossette et al., 2002; Fisher, 2004; Krampfl et al., 2005). Variants in the *GABRB3* gene, including *GABRB3* p.(Pro11Ser), p.(Ser15Phe) and p.(Gly32Arg), were all identified to cause reduced surface expression from hyperglycosylation (Tanaka et al., 2008), but later, receptors containing the p.(Gly32Arg) variant were also reported to have impaired gating (Gurba et al., 2012).

### 4.4 Summary

The early familial studies clearly demonstrated that genetic variants in GABA_A_ receptor subunits are associated with epileptic phenotypes. In most cases, the variants were reported to cause loss of receptor expression on the cell surface leading to reduced GABA‐elicited current amplitudes. Impaired receptor activation was also noted for some missense variants and dominant‐negative suppression of receptor expression was additionally proposed for protein truncating nonsense variants. Nevertheless, conclusions of the relative severity of the phenotypes associated with nonsense to missense variants were necessarily speculative in these studies due to both the low numbers of variants and patients, and the heterogeneity in clinical phenotypes within families carrying the same variant.

## 5 AN OVERWHELMING LOSS‐OF‐FUNCTION THEME IN GABA_A_ RECEPTOR VARIANT‐ASSOCIATED GENETIC EPILEPSIES

By the early 2010s, the cost of next‐generation sequencing techniques decreased dramatically, which allowed high‐throughput sequencing of affected patients, their parents and other family members. Sequencing and comparing the genomic sequences of affected and unaffected individuals thus became routine and resulted in the identification of a great number of de novo as well as familial variants in the GABA_A_ receptor genes.

### 5.1 De novo *GABR* subunits variants

The first reports of epilepsy‐associated de novo variants included variants within the GABA_A_ receptor subunit encoding genes, *GABRA1* and *GABRB3* (Allen et al., 2013). Soon thereafter, it became clear that genes such as *GABRB3* were responsible for a sizeable number of de novo likely pathogenic variants. Interestingly, these were associated with a broad spectrum of epilepsies that included severe DEEs, which differed significantly in clinical presentation to the prior reports of familial CAEs and GEFS+ (Hernandez et al., 2017; Janve et al., 2016; Le et al., 2017; Møller et al., 2017; Papandreou et al., 2016; Trump et al., 2016). While treatment with GABAergic enhancers was reported to improve clinical outcomes in some of these patients (Zhang et al., 2017), adverse drug reactions were also being reported in other instances, for example with the GABA‐transaminase inhibitor vigabatrin (Kothur et al., 2018; Papandreou et al., 2016). These observations suggest fundamental differences between the effects of the familial variants and some of the newly discovered de novo variants.

### 5.2 Loss‐of‐function traits

Functional analysis of receptors containing the newly discovered *GABRB3* variants presented a universal common theme of impaired receptor expression and/or activation. For instance, the maximum GABA‐elicited current amplitudes of receptors containing p.(Val37Gly), p.(Tyr184His), p.(Leu256Gln), p.(Tyr302Cys) or p.(Arg111*) variants were all significantly reduced in two‐electrode voltage clamp electrophysiological experiments (Møller et al., 2017). This loss of current was accompanied by a significant reduction in GABA sensitivity for the p.(Tyr184His) and p.(Tyr302Cys) variants. Whole‐cell electrophysiology recordings of receptors containing *GABRB3* p.(Asn110Asp), p.(Asp120Asn) and p.(Glu180Gly) variants reported either reduced current amplitudes or reduction in single channel burst duration (Janve et al., 2016). Furthermore, the *GABRB3* p.(Asn328Asp) and p.(Glu357Lys) variants were reported to impair synaptic clustering in neuronal cells (Shi et al., 2019), and the *GABRB3* p.(Leu170Arg) and p.(Ala305Val) were reported to reduce surface expression among other properties (Hernandez, Zhang, et al., 2017).

A similar scenario was observed for other subunit types, where loss of surface expression or impaired channel activity was reported. Receptors containing the *GABRG2* p.(Ala106Thr), p.(Ile207Thr), p.(Pro282Ser), p.(Arg323Trp) and p.(Phe343Leu) variants were all reported to reduce cell surface expression or impair receptor gating to varying degrees (Shen et al., 2017). The *GABRG2* p.(Gly257Arg) variant was reported to cause reduced palmitoylation and surface expression while the p.(Arg177Gly) variant was reported to cause impaired cell surface expression (Reinthaler et al., 2015; Todd et al., 2014). Receptors containing the *GABRB2* p.(Ile246Thr), p.(Pro252Leu), p.(Val282Ala) and p.(Ile288Ser) variants were likewise all reported to have reduced maximum GABA‐elicited currents when evaluated electrophysiologically (El Achkar et al., 2021).

### 5.3 Mixed functional effects

Although most variants were reported as loss‐of‐function, several groups reported variants that did not fall neatly into this consensus. A *GABRA5* p.(Val294Leu) variant was reported with a 10‐fold increase in GABA sensitivity, but also displayed increased desensitisation properties (Butler et al., 2018). Furthermore, variants that increase GABA sensitivity besides reducing current amplitudes were reported in *GABRA1, GABRA3, GABRB2* and *GABRB3* indicating this as a common phenomenon (El Achkar et al., 2021; Hernandez, Zhang, et al., 2017; Niturad et al., 2017; Steudle et al., 2020). Despite the evidence that the spectrum of GABA_A_ receptor variants displayed divergent functional outcomes, loss‐of‐function characteristics such as increased desensitisation and reduced surface expression (de facto loss‐of‐function) were being offered as preferred explanations as the cause of epilepsy in all these cases.

### 5.4 Summary

By the end of the 2010s, both familial and de novo GABA_A_ receptor variants had been regularly observed in many subunit types and a strong consensus had formed in the scientific community that epilepsy‐associated variants in GABA_A_ receptor subunits lead to an overall loss‐of‐function. Such loss‐of‐function traits were then used to provide support for a likely pathogenic role of the variants.

## 6 EMERGING QUESTIONS AND CONUNDRUMS

The problem with the simple generalist view that GABA_A_ receptor variants always cause loss‐of‐function is that it does little to explain the relationships between the changes in receptor properties and the wide spectrum of clinical phenotypes. While it was speculated that the phenotypic severity depends on the degree of loss‐of‐function (Qu et al., 2021), no clear evidence for such an association was provided. Thus, despite a constant increase in the number of functional results for new variants, the one size fit all loss‐of‐function theme raises as many questions as it answers.

### 6.1 Can loss‐of‐function create vastly divergent severe phenotypes?

If the phenotypic severity is dependent on the degree of loss‐of‐function caused by a variant, then the most severe forms of DEE must have a high degree of loss approaching 100%. However, while the term DEE is used for many patients with GABA_A_ receptor variants, the DEE umbrella covers a range of severe syndromes that have widely differing phenotypic characteristics and natural history. Patients with DS have substantially different disease manifestations from patients with West syndrome, and while some of these patients might respond favourably to ASMs, others do not. In fact, if all variants cause loss‐of‐function GABA_A_ receptors, then it would be expected that ASMs that enhance GABAergic signalling are beneficial in most if not all affected patients, yet this is not the case. While GABAergic enhancers show good effect in some patients, severe adverse effects can occur in other patients (Kothur et al., 2018; Papandreou et al., 2016). This raises the question of how a similar functional outcome (~100% loss) in a single subunit gene can lead to patient phenotypes that differ markedly on key clinical parameters.

### 6.2 Does impaired cell surface expression in vitro portray haploinsufficiency in vivo?

Many cases of null (protein truncating or splice‐site altering) variants have been reported for the *GABRA1*, *GABRB3* and *GABRG2* genes (Absalom et al., 2020; Jansen et al., 2006; Johannesen et al., 2016; Kananura et al., 2002; Lindy et al., 2018; Møller et al., 2017; Retterer et al., 2016; Zhang et al., 2017). In most cases, these can safely be considered as loss‐of‐function variants, whereby either nonsense‐mediated decay prevents the subunit from being translated, or a dysfunctional subunit is synthesised that fails to integrate into a functional receptor complex. Such loss‐of‐function variants would therefore lead to a de facto reduction in the combined gene output for two alleles (haploinsufficiency). At the same time, functional analysis suggested that a number of missense variants cause trafficking defects and reduced cell surface expression of functional GABA_A_ receptors (Fu et al., 2022; Hernandez et al., 2021; Lorenz‐Guertin et al., 2018). Hence, it would be expected that the phenotypes for patients with nonsense variants resemble those of patients with missense de novo variants that cause trafficking defects, as both are predicted to lead to a deficit of receptors in the synapse. However, this is not the case and while nonsense variants are typically inherited and can lead to mild (e.g., febrile seizures) or no phenotypes, missense de novo variants typically lead to more severe phenotypes (Absalom et al., 2022). This raises the question whether impaired cell‐surface expression observed in an in vitro setting reflects a haploinsufficiency scenario in a patient.

### 6.3 Have functional analysis provided reproducible and meaningful results?

As mentioned above, the first report of an epilepsy‐associated GABA_A_ receptor variant was the *GABRG2* p.(Lys328Met) variant (Baulac et al., 2001). Curiously, there have been many different explanations for how this variant causes loss‐of‐function including: (i) reduced current amplitudes (Baulac et al., 2001); (ii) temperature‐dependent trafficking defects (Kang et al., 2006); (iii) increases in synaptic decay rates (Eugène et al., 2007); and (iv) alterations in the membrane dynamics such that elevated temperature leads to the release of receptors from the synapse (Bouthour et al., 2012). While all these different explanations in themselves may represent plausible mechanisms, the diversity of these findings hardly appears consistent with a single missense variant in a single γ2 subunit of a pentameric receptor. Furthermore, several of the studies present conflicting information; for example, reduced current amplitudes were observed in one (Baulac et al., 2001) but not another study (Eugène et al., 2007). This raises the question of whether all observed effects caused by variants can be reproduced, and which if any of them are responsible for patient phenotypes.

### 6.4 Are some findings false positives?

As next‐generation sequencing became routine, the speed by which not only patient but also control population DNA was sequenced increased tremendously. In the context of interpreting genetic variation, this revolution led to two important developments. First, it became clear that there is a large degree of genetic variation in the general population where up to 2000 variations can be expected in a single person following whole genome sequencing (Auton et al., 2015). To aid in identifying whether genetic variants are likely to be benign or pathogenic, specific guidelines were developed by the American College of Medical Genetics and Genomics (ACMG) (Richards et al., 2015). Second, it became possible to assemble DNA information from large cohorts of the general population into databases. For example, public databases such as the genome aggregation database (gnomAD) provide a wealth of information regarding genetic variability in the general population, and if a genetic variant is seen multiple times in gnomAD, it is likely a benign polymorphism.

Considering this new information, it appears that numerous genetic variants that were previously presumed pathogenic and were shown to have significant loss‐of‐function effects in functional assays are in fact likely to be benign. For example, the *GABRA5* p.(Pro453Leu) and *GABRB3* p.(Glu357Lys) variants were proposed to cause significant alterations in channel gating characteristics or a reduction in receptor surface expression levels (Hernandez et al., 2016; Shi et al., 2019). However, these specific variants have now been observed in 7–8 individuals in gnomAD, which suggest that they are unlikely to be monogenic causes of severe disease and more likely benign polymorphisms in the general population. This is corroborated by a recent study, where the inherited *GABRB3* p.(Glu357Lys) variant did not cause any functional alterations and was concluded to be benign (Absalom et al., 2022). Thus, some previous claims regarding pathogenicity of variants may represent false positives, which raises the question whether the methodologies used to determine the functional outcome of variants were/are sufficiently rigorous.

### 6.5 Summary

By 2020, it was obvious that functional analysis efforts had failed to provide a good explanation for the confusingly variable display of phenotypes seen in patients with GABA_A_ receptor variants. A likely reason for this is that there was no consensus regarding how to perform functional analysis and which parameters to investigate. While different research teams reached the same conclusion that GABA_A_ receptor variants cause loss‐of‐function, this was often based on non‐comparable parameters. Furthermore, as genomic sequencing became routine, it emerged that false positives were contributing to the overall confusion.

## 7 GABA_A_ RECEPTOR VARIANTS CAN CAUSE BOTH GAIN AND LOSS OF FUNCTION

The first study attempting to directly link gain‐of‐function characteristics to clinical outcomes identified in patients with GABA_A_ receptor variants was published in 2020. In this case report, two patients with the *GABRB3* p.(Glu77Lys) and p.(Thr287Ile) variants displayed a severe hypersensitive reaction to vigabatrin treatment (Absalom et al., 2020). Vigabatrin is a GABA‐transaminase inhibitor that indirectly causes increased concentrations of GABA in the synapse. Electrophysiological analysis revealed that both variants conferred receptors with increased GABA sensitivity with no significant associated loss‐of‐function traits.

At the same time, two other *GABRB3* variants, p.(Leu284Met) and p.(Leu284Pro), were reported without functional evaluation (Mierzewska et al., 2021). This leucine residue is located within the ion channel pore and is fully conserved across all Cys‐loop receptor subunits (known as Leu‐9′). Earlier extensive site‐directed mutagenesis at various Cys‐loop receptors has shown the critical function of this residue in stabilising a closed receptor state and that a mutation of this residue typically causes gain‐of‐function (Chang & Weiss, 1999; Groot‐Kormelink et al., 2004). Hence, previous findings suggest that these two central leucine residue variants are also likely to cause gain‐of‐function.

Based on these observations, it seemed clear that the loss‐of‐function theme for GABA_A_ receptor variants was at best incomplete. To unravel this conundrum, we performed systematic analysis on larger patient cohorts using a standardised set of functional parameters and coupled these with detailed patient phenotypic data.

### 7.1 *GABRB3* cohort study

For the *GABRB3* gene, a large cohort comprising 85 individuals harbouring 63 variants was collected along with detailed phenotypic information (Absalom et al., 2022). Functional analysis revealed that 44 of the 54 missense *GABRB3* variants showed significant changes in receptor sensitivity to GABA when compared with the wild‐type receptor. These findings present support for a likely pathogenic or pathogenic role of these variants according to the ACMG guidelines. The remaining 10 variants did not significantly alter the investigated functional parameters, which supports classifications as variants of unknown significance or likely benign to benign. Surprisingly, 20 of the 44 variants with significant functional alterations displayed increased sensitivity to GABA, which constitutes a gain‐of‐function trait. The remaining 24 variants displayed decreased sensitivity to GABA, which constitutes a loss‐of‐function trait. Hence, functional analysis of a large set of *GABRB3* variants revealed that ~40% displayed gain‐of‐function, ~40% displayed loss‐of‐function and the remaining ~20% were either benign or could not be resolved with the standardised set of functional parameters.

When comparing the phenotypes of patients with gain‐of‐function to those with loss‐of‐function *GABRB3* variants, clear differences emerged. A striking clinical difference was the age of seizure onset, where the median age of the first seizure was 2.5 months for the gain‐of‐function cohort, but 11 months for the loss‐of‐function. Moreover, patients with gain‐of‐function variants typically presented with focal seizures, whereas patients with a loss‐of‐function presented with febrile and/or other generalised seizure types. The severity of intellectual disability was considerably worse, and the prevalence of hypotonia or microcephaly was significantly higher for patients with a gain‐of‐function variant. Notably, significantly better treatment outcomes were often identified in patients with loss‐of‐function variants, where drugs designed to enhance GABAergic activity were more likely to alleviate seizures, showing the importance of this knowledge for clinicians diagnosing and treating these conditions (Absalom et al., 2022).

### 7.2 *GABRD* cohort study

While patients with variants in the *GABRD* gene encoding the δ subunit are less frequent, a cohort of 10 patients harbouring seven variants was collected. δ‐subunit‐containing receptors belong to the group of extrasynaptic receptors and typically assemble with the α4 or α6 subunits (Ahring et al., 2022). Of the seven *GABRD* variants investigated, five displayed significant alterations relative to the wild‐type receptor and of these, four displayed gain‐of‐function traits and one displayed loss‐of‐function traits. Six patients with gain‐of‐function variants shared common phenotypes: neurodevelopmental disorders with behavioural issues, various degrees of intellectual disability, generalised epilepsy with atypical absences and generalised myoclonic and/or bilateral tonic–clonic seizures. In contrast, the one patient carrying a loss‐of‐function variant had normal intelligence and no seizure history but has a diagnosis of autism spectrum disorder and suffers from elevated internalising psychiatric symptoms. The finding that gain‐of‐function traits in δ‐containing extrasynaptic receptors cause epilepsy was corroborated by a later observation of a gain‐of‐function α4 subunit variant (Ahring et al., 2022).

### 7.3 Summary

At the present time, only variants in the *GABRB3* and *GABRD* genes have been systematically evaluated on a cohort basis. It is evident, however, that both gain‐ and loss‐of‐function variants are observed in these cohorts and that gain‐of‐function leads to more severe phenotypes. Importantly, these observations are consistent with the wide range of phenotypes observed across all patients. Isolated case reports of variants in the *GABRA1* and *GABRA5* subunits with increased GABA sensitivity indicate that a similar scenario may also be present for these genes and perhaps all GABA_A_ receptor subunits (Butler et al., 2018; Steudle et al., 2020).

## 8 HOW CAN A VARIANT AFFECT GABA_A_ RECEPTOR FUNCTION?

**FIGURE 2:**
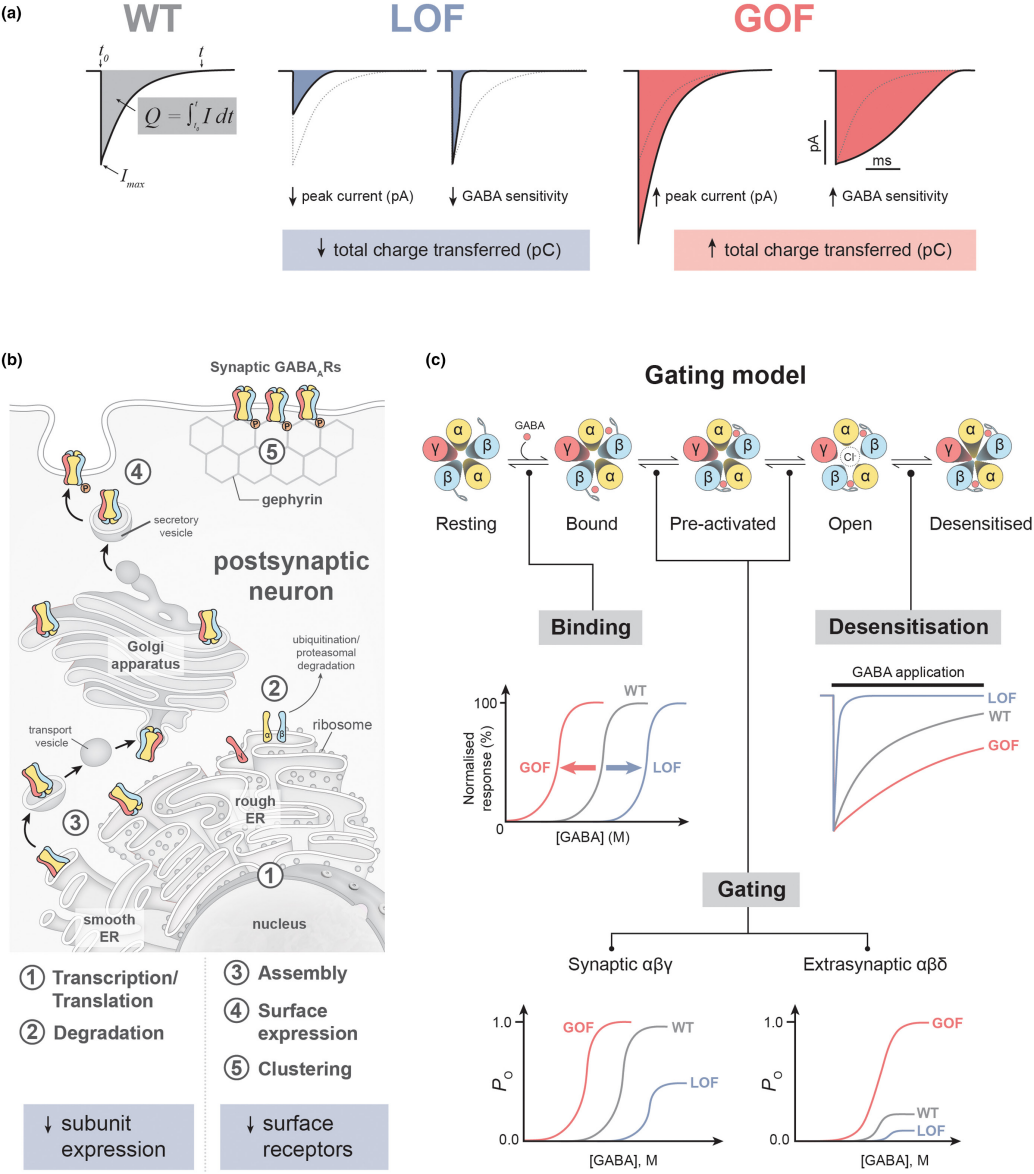
GABA_A_ receptor variants in epilepsy. (a) In a simplified conceptual model system, the impact of loss‐of‐function (LOF) and gain‐of‐function (GOF) GABA_A_ receptor variants on inhibitory postsynaptic currents can be assessed by measuring the total charge transferred (in picocoulombs, pC) during a synaptic GABA release event and comparing with wild‐type (WT). Quantifying the total charge transferred per event involves integrating current over time (i.e., calculating the area under the curve from *t*_0_ to *t*). (b–c) Molecular mechanisms of GABA_A_ receptor variants in epilepsy, whereby variants may lower the receptor density in a synapse or affect intrinsic receptor gating characteristics. (b) Impaired transcription or translation and nonsense‐mediated degradation may reduce the expression of receptor subunits, whereas issues with pentameric receptor assembly, endoplasmic reticulum (ER) retention, surface trafficking and postsynaptic clustering of GABA_A_ receptors may reduce the number of receptors delivered to a neuronal synapse. (c) A simplified gating model for GABA_A_ receptors. Cartoon models adapted from (Noviello et al., 2022). GABA_A_ receptor variants can affect the binding, pre‐activation, gating and/or desensitisation step(s) to change receptor function leading to GOF as well LOF.

Upon synaptic GABA release, GABA_A_ receptor‐mediated charge transfer in Coulombs determines the size and shape of the inhibitory postsynaptic potential. Given that GABA_A_ receptors are activated in multiple different contexts in vivo, a simplified conceptual model is required to meaningfully evaluate functional effects of variants. One such model is the integration of chloride current flow across a cell with a set membrane potential in response to a known GABA concentration that decreases over a set period (Figure 2a). Put simply, if the area under the curve is enlarged, then the variant is defined as gain‐of‐function, and if the area is diminished the variant is defined as a loss‐of‐function. Utilising area under the curve as an indicator, it is easy to understand how variants that increase or decrease the sensitivity of the receptor to GABA have significant impact and why they ought to lead to very different clinical outcomes. The first would lead to prolonged chloride flow for the duration of the event translating into increased area under the curve, while the other would lead to shortened time of chloride flow and decreased area under the curve.

As more GABA_A_ receptor variants are identified, it becomes imperative to develop a fundamental understanding of the underlying mechanisms by which they alter receptor properties towards gain‐ or loss‐of‐function. Furthermore, as most epilepsy‐associated GABA_A_ receptor variants are autosomal dominant, it is important to consider which are clinically relevant and minimal effect size limits as patients still have one normally functioning allele.

### 8.1 Variant‐induced changes to assembly and trafficking

Loss of cell surface expression of functional receptors has been observed with many GABA_A_ receptor variants in various in vitro heterologous overexpression assays. A variety of different variant types ultimately lead to this same outcome, and all have been categorised under the umbrella of loss‐of‐function variants (Figure 2b). These include protein truncating nonsense variants and splice site variants but also missense variants. Several potential and different explanations have been proposed for how a variant might reduce subunit expression. This includes:

(i) Impaired transcription or translation: In some instances, loss of subunit expression could arise from variants in promotor regions that cause lower transcriptional activity, or from nonsense and splice site variants that lead to nonsense‐mediated decay (Huang et al., 2012; Kananura et al., 2002; Tian & Macdonald, 2012; Urak et al., 2006).
(ii) Truncation and ER retention: In other cases, a truncated subunit protein might still be expressed. This may lead to dominant‐negative suppression, whereby the truncated protein form receptors with wild‐type subunits and accumulate in the ER. This reduces cell surface expression of not only the variant subunit but also other wild‐type subunits and can increase ER stress (Harkin et al., 2002; Johnston et al., 2014; Kang et al., 2009; Lachance‐Touchette et al., 2011; Maljevic et al., 2006).
(iii) Misfolding, degradation or ER retention of functional receptors: A substantial number of missense variants have been found to cause loss of GABA‐elicited current amplitudes. These variants potentially cause misfolding of the respective subunit leading to an aberrant pentameric receptor complex that is retained in the ER and/or undergoes degradation. Or they might directly affect the mechanisms responsible for protein maturation, trafficking towards the cell surface and clustering in the synapse (Cossette et al., 2002; Hirose, 2006; Huang et al., 2014; Lachance‐Touchette et al., 2011; Tanaka et al., 2008; Wallace et al., 2001; Wang et al., 2023).

Overall, the underlying proposed mechanisms for loss of cell surface expression can be seen as falling into two main groups: those that result in lower expression of a functional subunit and those that result in receptors that are arrested intracellularly. While the first resembles haploinsufficiency with missing subunits from one of the two alleles, the second is different as receptors are formed but potentially not delivered to the cell membrane. Nevertheless, these findings have all been proposed as pathophysiological mechanisms leading to epilepsy, under the assumption that loss of cell surface expression in vitro reflects loss of receptor density in neuronal synapses.

### 8.2 Variant‐induced changes to binding and gating properties

Other variants have been described to affect receptor gating properties. Variants that affect the binding or receptor activation processes will alter either the potency of GABA at the receptor and/or the intrinsic efficacy of the receptor when activated by GABA, which can cause gain‐ as well as loss‐of‐function traits. The kinetic mechanisms that govern receptor activation have been studied in detail (Castellano et al., 2022; Gielen et al., 2012; Keramidas & Harrison, 2010; Mortensen et al., 2004). While the precise kinetic steps continue to be debated, a minimum of four key steps are needed to describe receptor activity. These include GABA binding, pre‐activation, channel gating and receptor desensitisation (Figure 2c). While variants could potentially alter any of these four steps in the gating process, some lead to simpler outcomes than others. For example, variants that cause alterations within the GABA binding site may represent simple cases as they can increase or decrease the receptor sensitivity to GABA in isolation. However, both kinetic models and results from site‐directed mutagenesis have shown that variants that affect the pre‐activation or the channel gating steps have more complex effects and typically alter both GABA sensitivity and gating efficiency (Jatczak‐Śliwa et al., 2020; Szczot et al., 2014). Changes to the desensitisation step are also unlikely to occur in isolation but linked to changes in gating efficiency. Nevertheless, a commonality for all these four steps is that variants may incur either gain‐of‐function or loss‐of‐function traits or a combination thereof.

The subtype heterogeneity of GABA_A_ receptors further complicates studies of gating characteristics. For instance, a single de novo variant in a β3 subunit incorporates into both synaptic α1β3γ2 and extrasynaptic α4β3δ receptors. These receptors have different GABA affinities as well as different intrinsic open probabilities in response to GABA binding (different gating efficiency) that affect the way the variant will influence receptor properties (Brickley et al., 1999; Brickley & Mody, 2012; Luo et al., 2013). At γ2‐containing synaptic receptors, GABA is a low potency/high efficacy agonist, where large concentrations released at the synapse are required to activate the receptor but, when fully bound, the probability the receptor will be open approaches 100% (Mortensen et al., 2011). In this case, a gain‐of‐function variant that increases channel gating will primarily be observed to increase the GABA potency, as there is little room to increase the maximal GABA efficacy. At δ‐containing extrasynaptic receptors, however, GABA is a high potency/low efficacy agonist, where low concentrations of circulating GABA or synaptic spill‐over activate the receptors but, when fully bound, the probability of the receptor opening is perhaps less than 10% (Ahring, Liao, Gardella, et al., 2022; Liao et al., 2021). Here the same gain‐of‐function variant will increase both GABA potency and the maximal GABA efficacy (Figure 2c). These differences resemble how anaesthetics and neurosteroids exert different effects on different receptor subtypes (Ahring et al., 2016; Herd et al., 2007). Hence, the exact functional outcome of a variant depends on the receptor type studied.

### 8.3 Do all parameters translate into functional alterations in vivo?

With so many potential ways, a variant can cause functional alterations of GABA_A_ receptors, it is relevant to consider the relative contribution and significance of these. The difficulty with this is that it is not necessarily straightforward to predict how the various alteration types translate into functional changes of importance for a patient.

Alterations to receptor binding, pre‐activation and gating steps are likely to have a high degree of translation from in vitro observations to neuronal receptors. At the simplified conceptual level of the total charge transferred from the release of a single GABA vesicle, it is reasonably straightforward to predict the outcomes. For example, a change to GABA sensitivity in vitro would likely result in a similar change for receptors in vivo and thereby cause increased or decreased area under the curve. However, while changes to the desensitisation step would also be expected to alter receptors in vivo, it is harder to predict what the outcome of this will be. Changes to desensitisation properties may only have minor effects at synapses that are functioning within normal firing parameters, where there is adequate time for receptors to recover from desensitisation. That said, desensitisation changes might have a significant impact under a pathological condition with uncontrolled continuous neuronal firing such as during an epileptic attack. Here, increased desensitisation characteristics could force receptors into the desensitised state thereby acting to attenuate the issue. The reverse might also occur where decreased desensitisation could render a larger fraction of receptors available to continuous firing thereby exacerbating the condition.

Alterations to assembly and trafficking processes are notoriously complex to evaluate. It appears reasonable to assume that variants that substantially hinder subunit expression or normal protein folding and membrane assembly of a single subunit would lead to a loss of pentameric receptors containing this subunit in a neuron. However, trafficking issues with fully assembled and functional receptors or dominant negative effects observed in heterologous expression systems may or may not translate into loss of receptor density in neuronal synapses. One reason for this is that compensatory mechanisms such as upregulation may alleviate certain types of issues but not others. Another reason is that neurons are known to assemble more receptors internally than are needed at their synapses (receptor reserve), meaning that the receptor density in a neuronal synapse may not be significantly affected despite the slower trafficking characteristics. This is underscored by the observation that the early familial nonsense or missense variants, that were described as dominant negative or resulted in reduced surface expression, were not fully penetrant in the respective families (Audenaert et al., 2006; Harkin et al., 2002; Kananura et al., 2002; Kang et al., 2009; Wallace et al., 2001). Hence, it is difficult to assess the degree by which trafficking defects observed in an in vitro overexpression system translate into a reduction in number of receptors in neuronal synapses and this could differ substantially from case to case.

### 8.4 Summary

A deep understanding of how variants affect GABA_A_ receptors is important as this knowledge enables us to categorise variants reliably and to group patients into like‐for‐like cohorts when performing genotype–phenotype correlation analysis. It is, however, clear from the above that variants can introduce alterations to normal GABA_A_ receptor function in multiple ways. Some of these alterations may lead to gain‐of‐function others to loss‐of‐function and some may even a lead to a mixture thereof. Furthermore, although some of these alterations may display clear translation into the human setting, this is not universally the case, and it can be difficult to decipher the relative importance. Therefore, it would be illogical to assume pathogenicity for all types of differences observed between a variant and the wild‐type receptor in an in vitro assay. To resolve which functional differences are important, we need to integrate the information obtained from functional assays with clinical information from large cohorts of patients to determine the clinical correlate for each parameter.

## 9 MOVING FORWARD: PRACTICAL GUIDELINES FOR FUNCTIONAL ANALYSIS AND INTERPRETATION OF GABA_A_ RECEPTOR VARIANTS

**FIGURE 3:**
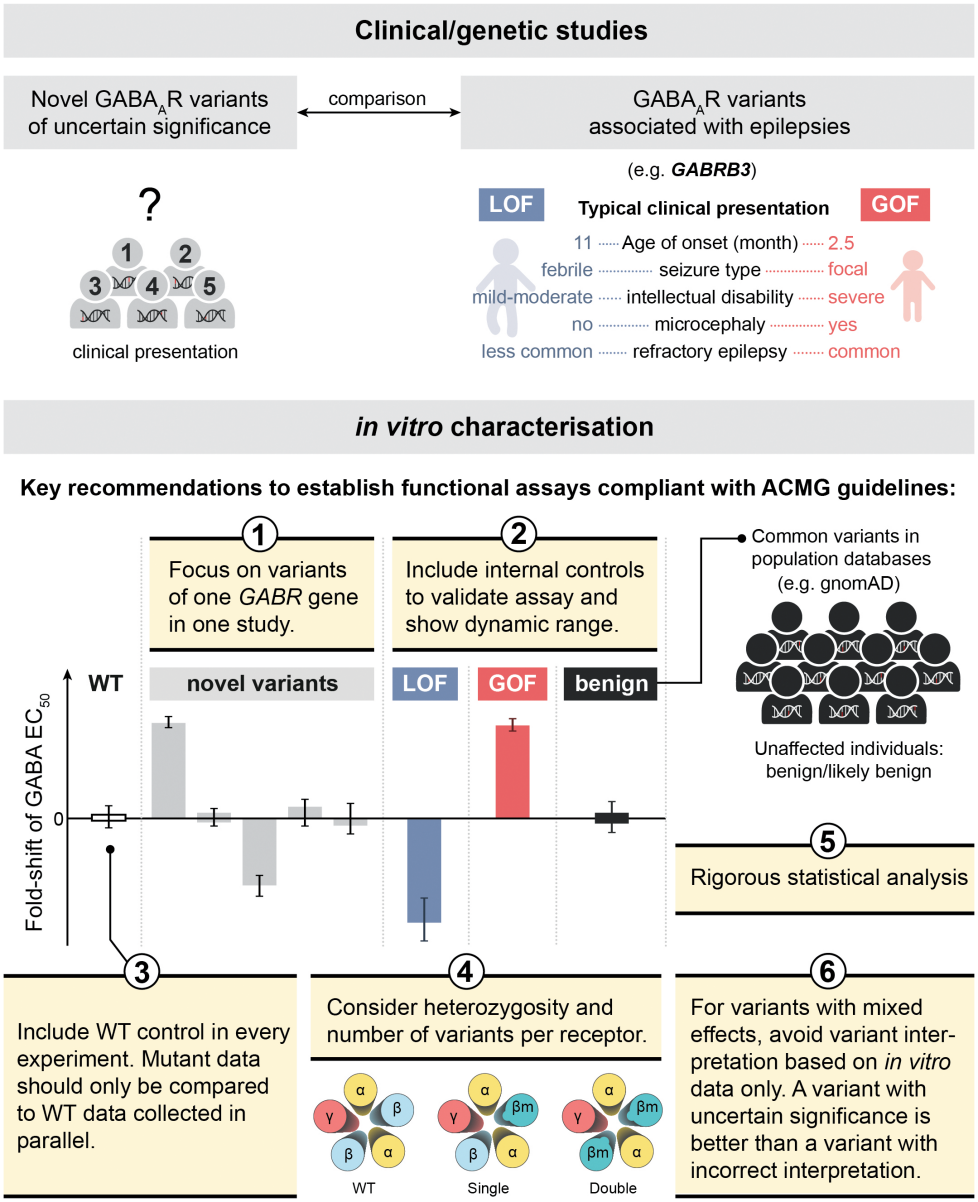
Best practice guidelines for the use of functional assays in the interpretation of GABA_A_ receptor variants. To enable strong support for a benign or pathogenic role of novel genetic variants, these should be evaluated in a well‐established functional assay that is compliant with the ACMG guidelines (Brnich et al., 2019). This cartoon provides some key recommendations for ensuring that an in vitro electrophysiological assay displays the required discriminatory power for analysing novel GABA_A_ receptor variants. *GABRB3* phenotypic information adapted from (Absalom et al., 2022). WT, wild‐type; LOF, loss‐of‐function; GOF, gain‐of‐function.

For early small‐scale analysis of GABA_A_ receptor variants, the task consisted of identifying altered functional parameter(s) that could explain the patient phenotype. However, such a simple approach is both error‐prone and inadequate. Instead, it is important to interpret functional and clinical data combined within the framework of the ACMG guidelines. In these guidelines, one important type of evidence is the effect of a variant determined by a “well‐established” functional assay, and this can provide a pathogenic‐strong or benign‐strong support for the impact of the variant (rule codes PS3 and BS3). To enable such interpretation within a clinical scope, the functional assay must be validated (Brnich et al., 2019). Full assay validation requires that an extensive set of both experimental controls as well as clinical validation controls are tested alongside novel variants. Such rigour may not always be possible for research laboratories, nevertheless, to avoid false positives is important to adhere to as many of the recommendations as possible (Figure 3). For the functional analysis of GABA_A_ receptor variants, the following points should be considered.

### 9.1 Cohort studies and focussing on one gene at a time

When next‐generation sequencing was at its infancy, it made sense to aggregate variants in genes encoding different GABA_A_ receptor subunits into a single study due to the limited numbers. This is no longer necessary and can also add complexity to the study design thereby increasing the risk of erroneous conclusions. A common issue with functional characterisation of GABA_A_ receptor variants is that the subtypes of interest are typically ternary receptors, yet upon co‐expression, some of the individual subunits can readily assemble into alternative functional receptor combinations leading to expression of non‐uniform receptor populations (e.g., binary α1β2 along with ternary α1β2γ2) (Liao et al., 2021). Substantial assay optimisation is often required to ensure that experiments with variants are performed using comparable and uniform populations of receptors. Therefore, it is good practice to reduce experimental complexity and optimise experimental conditions to study numerous variants in one gene at a time.

### 9.2 Positive, negative and benign controls

When performing experimentation on a set of novel variants, it is good practice to include clinical validation controls besides the standard wild‐type control (Brnich et al., 2019). These controls are meant to include known pathogenic and benign genetic variants that lead to both positive, negative, and benign outcomes for the specific functional parameter being investigated. If possible, these controls should be blinded to the investigator and there are two reasons for why they are so important. First, for the functional data to comply with the framework of the ACMG guidelines, it is imperative to document that the assay can robustly identify gain‐of‐function, loss‐of‐function and benign variants. Second, there are many benign variants in the general population as recorded in the gnomAD and some of these may show minor alterations in receptor function. Testing such benign variants help to determine how much of a functional change can be tolerated within the normal spectrum.

Hence, in an experimental scenario where positive and negative controls are not identified correctly, the functional evaluation may not be valid and therefore no conclusions should be drawn. Furthermore, to label a variant as pathogenic requires not only that it differs in function from a canonical wild‐type receptor but that it also differs to benign variants in the general population.

### 9.3 Parallel experimentation

Irrespective of the assays used, every step in the assaying process can introduce variability and it is therefore good practice to perform all experiments in parallel with the wild‐type receptors control. For example, unforeseen variability might occur during the process of making new variant cDNA constructs, which could make these unsuitable for comparison with wild‐type cDNA constructs that were produced at an earlier time point. Likewise, intraday variability can be significant during functional experimentation making it inappropriate to compare data of variant receptors with data of wild‐type receptors that were performed at a different time point. Even though parallel experimentation means continuous repetition of control experiments on every experimental day, comparisons should only be made for data performed in parallel.

### 9.4 Single and double variant receptors

Ternary receptor such as α1β2γ2 contain two α1, two β2 and one γ2 subunit. For α and β subunit variants, this means that receptors can contain one or two variant subunits. Since the effects of variants are typically additive, this means that receptors with two variant subunits will show a larger detriment than those with only one variant subunit. Assuming normal Mendelian distribution, double‐variant receptors only constitute 25% of the total population of receptors in the human brain, while single‐variant receptors constitute 50%. To understand how most receptors behave, it is therefore important to investigate single‐variant receptors and studies focussing solely on double‐variant receptors may reach incorrect conclusions. For example, if a variant promotes trafficking issues, this could lead to a scenario where the double‐variant receptors are fully retained intracellularly while single‐variant receptors are still efficiently expressed at the cell surface. In such a case, in vitro findings for double‐variant receptors may be irrelevant as single‐variant and wild‐type receptors are the ones present at the synapse.

An elegant methodology for evaluating single‐variant receptors is to use concatenated cDNA constructs (Absalom et al., 2019; Ahring et al., 2022; Liao et al., 2019, 2021). Here, all five subunits necessary to form a single GABA_A_ receptor are assembled into one cDNA construct and deliver a fusion protein constituting a complete receptor when expressed. This methodology allows unique control of the number and placement of variant subunits within the GABA_A_ receptor complex. Furthermore, it eliminates the issue with non‐uniform receptor populations and polluting binary receptors.

### 9.5 Statistical analysis

There are several good practices to follow with respect to statistical analysis. In general, power analysis should be performed to calculate the minimum number of biological replicates necessary to show statistical effect. This number will vary greatly depending on the parameter investigated but generally n = 5 should be seen as the absolute minimum (Curtis et al., 2022). Next, it is imperative to ensure that the final datasets for variants and controls encapsulate the true variability in the dataset for the parameters analysed. While this might seem trivial, many commonly used statistical programs (e.g., GraphPad Prism) do not report the real standard deviation values for non‐linear regression results. With respect to statistical tests, it is important to use parametric and non‐parametric ANOVAs appropriately for functional parameters and Fisher's Exact test or non‐parametric t‐tests for clinical parameters. Finally, to limit the risk of false positives, it is generally recommended to use conservative p‐value thresholds such as *p* < 0.001 or *p* < 0.0001.

### 9.6 Variants with mixed effects

As noted above, mixed gain‐ and loss‐of‐function traits have been observed for GABA_A_ receptor variants in several cases (Butler et al., 2018; Hernandez, Zhang, et al., 2017; Niturad et al., 2017; Steudle et al., 2020). In these cases, the question arises which effect if any is more important and ends up dominating. Attempting to answer this question purely based on in vitro data quickly becomes speculative, and instead, the patient phenotype should be the arbiter in these cases. By aligning the patient phenotype with data for known validated clinical controls an answer might be reached. For example, if the patient phenotype has all the hallmarks of other patients with established gain‐of‐function variants, then the gain‐of‐function trait is likely dominating. Vice versa, if the patient phenotype resembles that of other patients with loss‐of‐function variants, then the loss‐of‐function trait is likely dominating. If the patient phenotype does not match any of these two groups, then the mixed effects of the variant are likely both contributing significantly to the phenotype, and this variant should possibly be categorised as a mixed gain‐ and loss‐of‐function variant.

### 9.7 Summary

The above presents some of the important points to consider when performing functional analysis of GABA_A_ receptor variants to comply with the framework of the ACMG guidelines. This does not constitute a comprehensive list, and there will likely be other aspects to consider depending on the specific assay. The key point to note is that false positives should be avoided wherever possible and that not assigning an effect for a variant is better than assigning an incorrect effect. This is particularly important when dealing with functional alterations of a variant that may not display clear translation into the human setting and/or where there are no established clinical validation controls. Finally, it is important to recognise that these above points likely exclude many historical publications from interpretations within the guidelines. In these cases, ACMG codes such as PS3 and BS3 should not be applied, meaning that the functional data should not be used to provide strong support for pathogenicity of the variant. An inference of this is that utmost caution should be used when attempting to compile functional data from numerous sources.

## 10 BEYOND SIMPLE CONCEPTUAL MODELS

In a commonly used and convenient simple theoretical network model, brain activity is tightly regulated by the balance between excitatory and inhibitory synaptic activity (Figure 1a). The GABAergic system provides the main inhibitory tone and decreased or increased GABAergic activity will tilt the balance towards more or less overall brain excitation. As presented in the previous sections, another simple conceptual in vitro model of total charge transferred in response to a defined GABA stimulus (Figure 2a) is proving very useful in categorising variants as gain‐ or loss‐of function and aligning these results to the divergent clinical phenotypes observed. While it may be tempting to consider these two models in combination, extrapolating from the simple in vitro model to the simple network model without careful consideration of the many different contexts where GABA_A_ receptors are activated in vivo is unlikely to explain how gain‐ or loss‐of‐function variants affect fundamental neurological processes.

First, at the cellular level, the total charge transferred is dependent on many factors including the chloride gradient, magnitude of tonic inhibition and interplay between the frequency of GABA release and the rate of transporter‐mediated chloride efflux (Figure 1b). For instance, whether GABAergic transmission is excitatory or inhibitory depends on the resting chloride gradient, and if chloride transport mechanisms cannot cope with excess GABAergic transmission, then high‐frequency stimulation will reduce the inhibitory capacity of GABA_A_ receptor activity, or potentially even shift it from inhibitory to excitatory. Secondly, at the level of neuronal circuits, the complexity is magnified even further where cellular inhibition can result in either an increase or decrease in overall network excitability. For instance, VIP^+^ neurons can preferentially inhibit SOM^+^ neurons, leading to disinhibition of pyramidal neurons, increasing the probability of action potential firing (Figure 1a). Thus, at disinhibitory circuits, gain‐of‐function variants will be expected to enhance downstream neuronal activity and loss‐of‐function variants to inhibit them. Finally, the complex interplay between synaptic and extrasynaptic inhibition, GABAergic activity in different brain regions and compensatory mechanisms as the patient develops cannot be predicted from extrapolations of the simple in vitro model to a simple network model.

Because of the complexity at the cellular and neuronal levels, the molecular classifications of gain‐ and loss‐of‐function should not be unthinkingly extrapolated to simple network models and such attempts will likely lead to wrong conclusions. Instead, an understanding of the precise pathomechanisms underlying receptor variants will require incorporation of all the different contexts of GABAergic activity. As it appears counterintuitive that both gain‐ and loss‐of‐function variants can lead to epilepsy, further research into these mechanisms is greatly needed to develop sophisticated models that explain these aspects of the disorder. In the meantime, associating clinical phenotypes with the molecular function brings us a long way in understanding the clinical distinction between gain‐ and loss‐of‐function variants.

### 10.1 Future perspectives

In an ideal clinical scenario, the likelihood of pathogenicity, prognosis and best treatment options of a patient can be predicted upon identification of a variant in a GABA_A_ receptor gene. While this is a realistic goal, there are still considerable hurdles to be overcome. Functional evaluation of all GABA_A_ receptor variants within the ACMG framework is essential to develop the knowledge base required to prevent false positives and to correlate patient phenotypes with gain‐ or loss‐of‐function, or mixed functional effects. Key to this framework is the recognition that assay controls as well as clinical validation controls represent an essential part of the analysis process. When such a framework is implemented, it will become possible to assemble data from worldwide sources to significantly further our understanding of the pathophysiological mechanisms of GABA_A_ receptor variants. Only then can we reach a state, where we can determine the likely pathogenicity and predict the severity of epilepsy disorders in newly diagnosed patients.

Developing treatments for GABA_A_ receptor‐associated epilepsies represent a different set of hurdles. Several ASMs that enhance GABA_A_ receptor activity are already available, and novel agents to treat loss‐of‐function variants strictly via this pathway would need to be either significantly more efficacious or have less side effects. While benzodiazepines selectively enhance currents at γ2‐containing receptors, most of the current drugs have a low degree of receptor subtype selectivity limiting their use due to side effects such as sedation. Hence, drugs that selectively enhance GABAergic currents at receptors containing specific subunits would greatly aid a future precision medicine approach. In contrast, no current treatments reduce GABAergic inhibition, and it is likely that elegant precision medicine strategies will need to be developed for gain‐of‐function variants. Antisense oligonucleotide therapy holds promise for treatment of several different gain‐of‐function channelopathies that may also prove effective at GABA_A_ receptors, with the added virtue that only a specific receptor subunit is targeted. For patients with severe DEEs, alleviating the effect of comorbidities also remains a high priority, and precision medicine techniques targeted directly at the GABA pathway may prove effective at both seizures and the different comorbidities.

## AUTHOR CONTRIBUTIONS

Nathan L. Absalom, Susan X. N. Lin, Rikke S. Møller, Han C. Chua and Philip K. Ahring wrote and edited the manuscript; Han C. Chua prepared and created the figures; all authors revised the manuscript.

## CONFLICT OF INTEREST STATEMENT

The authors declare no conflicts of interest.

## Data Availability

Data sharing is not applicable since no new data were generated for this manuscript.
